# Analysis of inter-brain synchrony in group-based electroencephalography to assess task-dependent interactions

**DOI:** 10.3389/fnrgo.2026.1774423

**Published:** 2026-03-31

**Authors:** Alex Kennedy, Nathan Shields, Sean Farrell, Alejandro Lopez Valdes

**Affiliations:** 1 Department of Mechanical Manufacturing and Biomedical Engineering, School of Engineering, Trinity College Dublin, The University of Dublin, Dublin, Ireland; 2 Trinity Centre for Biomedical Engineering, Trinity College Dublin, The University of Dublin, Dublin, Ireland; 3 Department of Electronic and Electrical Engineering, School of Engineering, Trinity College Dublin, The University of Dublin, Dublin, Ireland; 4 Trinity College Institute of Neuroscience, Trinity College Dublin, The University of Dublin, Dublin, Ireland; 5 Global Brain Health Institute, Trinity College Dublin, The University of Dublin, Dublin, Ireland

**Keywords:** EEG, group task, hyperscanning, individual task, inter-subject correlation, neural synchrony, social interaction

## Abstract

**Introduction:**

Social interaction and cooperative behavior are inherent and important aspects of daily life. Neuroscience research has demonstrated that neural activity synchronizes during cooperative group behavior. Hyperscanning, a method of simultaneously recording neural activity from two or more subjects, allows insight into the underpinnings of neural dynamics.

**Methods:**

This study involves a triadic 24-channel EEG hyperscanning experiment, using a cooperative card game to elicit group interaction and cognitive puzzle games as individual control tasks. The study was split into two separate experiments. Experiment One, where two groups repeatedly performed experimental blocks and Experiment Two where 10 individual groups participated in one block, where an adversary was randomly introduced to determine if negative social behavior changed neural synchrony. After removing artefactual contributions of muscle and eyeblink components and task duration discrepancies that may affect the group's synchrony, the neural correlation between subjects was examined via Inter-Subject Correlation (ISC). Linear mixed-effect models were used to assess the magnitude of differences in ISC, unadjusted, and adjusted trial-duration.

**Results:**

Similar neural synchrony levels were observed in the group members in Experiment One (unadjusted: cooperative ISC = 0.286 ± 0.013, individual ISC = 0.267 ± 0.02, baseline ISC = 0.219 ± 0.008, duration-adjusted: cooperative ISC = 0.225 ± 0.015, individual ISC = 0.278 ± 0.017, baseline ISC = 0.23 ± 0.007) and Experiment Two (unadjusted and duration-adjusted: cooperative ISC = 0.186 ± 0.009, individual ISC = 0.177 ± 0.01, baseline ISC = 0.157 ± 0.005).

**Discussion:**

While no statistically significant differences were found between cooperative and non-cooperative tasks, task-based synchrony was higher than resting state synchrony. Furthermore, significantly higher brain synchrony was observed in cooperative tasks when there were no adversaries present in the group. This study highlights the importance of analysis parameters like the analysis time window and task contrasts avoiding similarities in cognitive demands when evaluating brain synchronization in naturalistic environments for group-based interactions.

## 1 Introduction

Humans are social creatures, and daily life involves communication and social interactions with other human beings. The importance of social interaction cannot be understated for mental health and numerous aspects of life, such as physical health and quality of life. The shaping of the mind and brain function is due to the continuous interaction with other humans (Hari et al., 2015). Still, understanding the neural activity that manages social behavior is minimal (Young, 2008). Social interaction can be perceived as an exchange between two or more neural systems linked together through behavior and sensory inputs (Kingsbury and Hong, 2020), involving numerous interactions and social behaviors such as cooperation, competition, negotiating, questioning, imitation and bargaining (Hari et al., 2015). The examination of neural activity during social interactions within the field of neuroscience has only begun to be investigated in the last few decades (Babiloni and Astolfi, 2014). Researchers studied groups of two individuals (dyads) to fill that void when examining social interaction, known as the “two-brain approach” (Konvalinka and Roepstorff, 2012). This method allows for investigating how each brain impacts the other during their interaction, examining their inter-brain dynamics in real-time, and providing insight into how information is exchanged between them. The subsequent measuring of brain activity from dyadic experiments using neuroimaging modalities such as electroencephalography (EEG), functional near-infrared spectroscopy (fNIRS) and functional magnetic resonance imaging (fMRI) allowed for quantifying the inner workings of neural activity, known as inter-brain mechanisms (Konvalinka and Roepstorff, 2012). This simultaneous recording of neural activity between subjects has been termed “hyperscanning” (Montague, 2002).

Social interactions usually include more than two people; therefore, dyadic groups limit obtaining as much information as possible. A viable method to explore social interaction is to consider a group of people as part of a connected system, where inter-brain dynamics between members could be examined to see their relation, known as the “multi-brain approach” (Kingsbury and Hong, 2020). Research has revealed that brains synchronize when performing a similar task, known as inter-brain synchronization (IBS), which needs to be decoded to fully disentangle the underlying neural dynamics among brains within a group. The neural mechanisms during social interaction in a group cannot be understood by examining the individual's activity; instead, by designing a precise experimental procedure involving multiple individuals interacting, with sufficient analysis methods to quantify the inter-brain interactions (Konvalinka and Roepstorff, 2012). The problems in quantifying inter-brain activity and interactions are not due to modality issues but the approach to correctly asking and answering the questions themselves. The study of social interaction is complicated as the environments in which observations and recordings occur are rarely entirely naturalistic. Additionally, it is unnatural that people interact with those they would not normally interact with, and they are likely to alter their behavior and interactions due to the presence of being recorded and observed (Kenny, 1996). Therefore, to replicate social interaction as closely as possible to the real world, close attention must be placed on making the experimental design as naturalistic as possible to accurately record an authentic representation of the interactions between a group of people.

Hyperscanning is the simultaneous acquisition of neural data from two or more subjects (Babiloni and Astolfi, 2014) and the term was first introduced in 2002 when two subjects played a game simultaneously in separate fMRI machines (Montague, 2002). Since then, the hyperscanning technique has been expanded to other modalities, such as fNIRS, EEG, and MEG (Hirata et al., 2014; Li et al., 2021). Different experimental procedures have subsequently been designed around EEG hyperscanning to obtain insights regarding the flow of information, synchrony, network properties, and neural dynamics (Czeszumski et al., 2020) from traditional hyperscanning with two subjects (i.e., dyadic hyperscanning) to newer experimental designs involving three subjects (i.e., triadic hyperscanning). EEG hyperscanning offers insights into the neural mechanisms of cognitive function and decision-making, which are fundamental aspects of social interaction (TenHouten et al., 2023). The availability of simultaneously recorded data from numerous participants opens up areas for new research and analysis, not only for how the neural activity is related to their behavior for each subject but also how the brain activity is related to the brain of the interacting partner engaged in the same task (Babiloni and Astolfi, 2014). Hyperscanning provides an ecologically valid way to study the inner workings of the social brain during social interactions (TenHouten et al., 2023).

IBS is a neural observation in which brain activity across subjects correlates across time under specific conditions. The idea is established on the notion that the temporary excitability states in the cortical region are indicated in the neural oscillations, which, when synchronized across individuals, might allow for the coordination of behavior or the sharing of cognitive function (Kingsbury and Hong, 2020; Koul et al., 2023). Synchronous brain behavior is essential in creating social ties, cues, and a collective social community (TenHouten et al., 2023). Components of neural activity are inscribed with sensory and behavioral cues which contribute to the dynamics at play during IBS, but other hidden aspects play an essential role (Kingsbury and Hong, 2020). A review paper investigating different hyperscanning modalities, paradigms, and experiments revealed that IBS has been identified to have a substantial effect in both the frontal and temporoparietal areas of the brain, with the maximum effect being discovered in the prefrontal cortex (Maslova et al., 2022). A separate review paper examining fNIRS hyperscanning studies demonstrated that IBS can be observed in the prefrontal cortex and supported the engagement of this region during cooperative behavior (Czeszumski et al., 2020). Additionally, hyperscanning studies have revealed that IBS plays a pivotal role in communication, cooperation, and attention, with IBS studies focusing on cooperative hyperscanning tasks.

**Figure 1:**
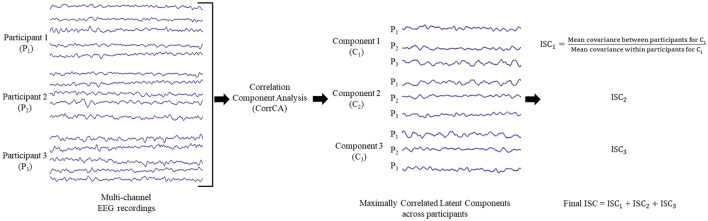
Inter-Subject Correlation (ISC) calculated using correlated component analysis (CorrCA). Multi-channel EEG data from each participant is recorded, and combinations of weighted components that are maximally correlated across participants are identified. These components are not spatially localized to individual electrodes or regions of the brain. The three maximally correlated components are retained, and the final ISC value is calculated as the sum of the between participant to within participant covariance ratios for these three components.

IBS studies have rarely been examined beyond a dyadic dynamic (Azhari et al., 2025), with only a few investigating triadic or, more in passive non-cooperative experiments (Cohen et al., 2018; Cohen and Parra, 2016), indicating a need to study beyond dyads for probing social interaction. Two hypotheses have been proposed to clarify the appearance of IBS during interactive decision-making in (Hu et al., 2018), the cooperative interaction hypothesis vs. the comparable task hypothesis, suggesting that IBS may originate from interaction between people and a predisposition to cooperate with others or the latter, that IBS may be reflected in the similarity of a common task. The experimental design must be able to differentiate between these hypotheses. IBS can be analyzed using inter-subject correlation (ISC), which exploits neural entrainment through experimental paradigms. As depicted below in Figure 1, ISC is an analysis method for elicited brain activity from tasks to determine spatiotemporal patterns which are reliable across subjects (Holroyd, 2022).

This study aimed to analyze a naturalistic experimental set-up that measured inter-brain synchrony during a three-person (triadic) group and individual task at a more comprehensive level to investigate and understand the complex mechanisms exhibited during group behavior across two experiments.

## 2 Materials and methods

### 2.1 Participants

Thirty-six (36) participants took part in this study, separated into two experiments (six in Experiment One and 30 in Experiment Two). All participants provided written consent; all were over 18 years old, had no neurological disorders and were asked not to consume alcohol, tobacco or caffeine within 12 h of the experiment. While we stipulated that participants should not know each other before commencing the experiments, as members of the student body of the same university, there was an unavoidable degree of familiarity among some of the participants, however, none had close friendships. Research ethics approval was sought and granted for both experiments by the School of Engineering Research Ethics Committee of Trinity College Dublin, The University of Dublin.

### 2.2 Experimental design

**Figure 2:**
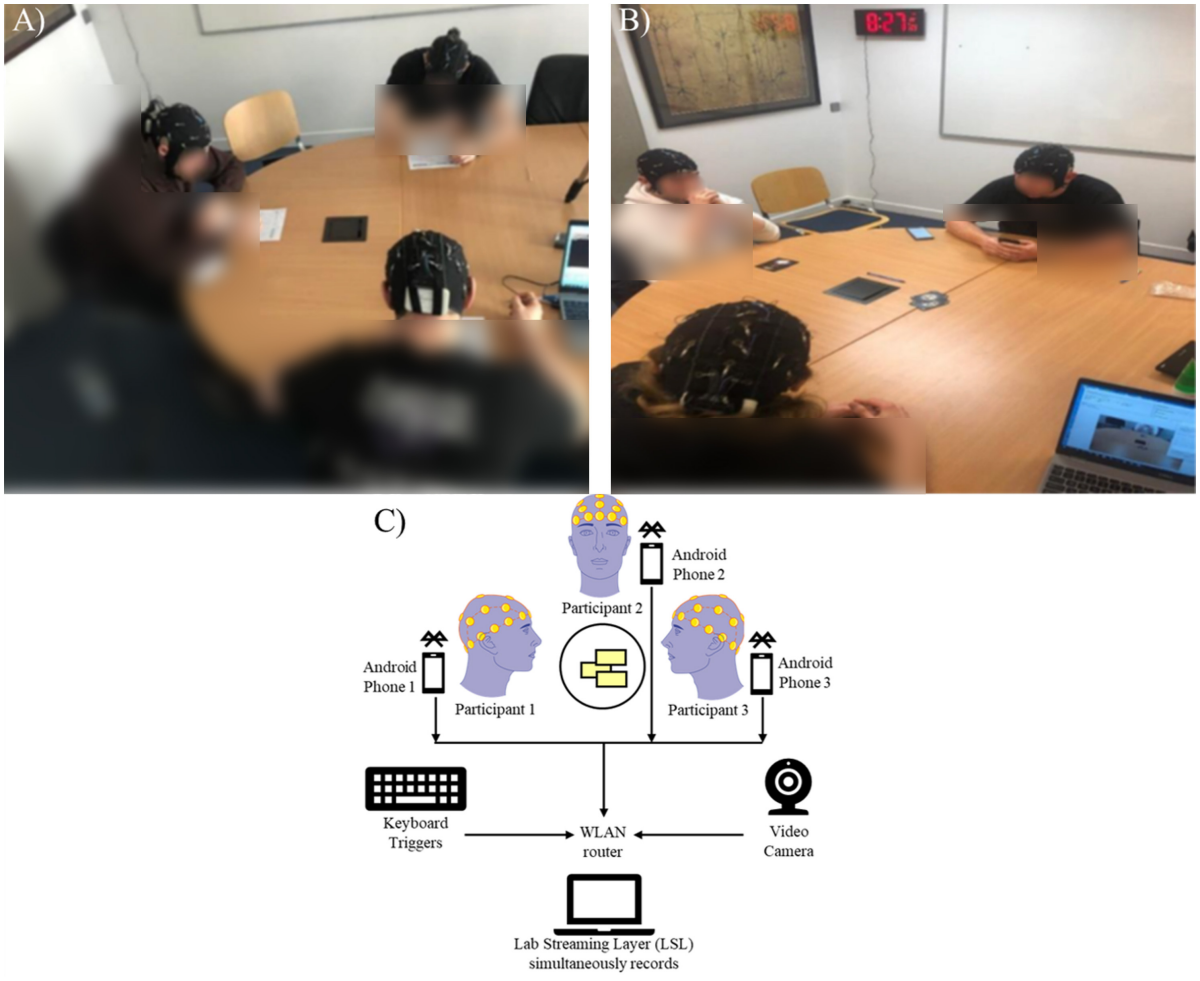
**(A)** The experimental set-up of the individual task depicts each of the three participants doing a unique task while their EEG signals are simultaneously recorded. **(B)** The experimental set-up of the cooperative task, depicting each of the three participants performing a cooperative task, the card game. **(C)** The experimental set-up schematic of the cooperative task, depicting the three subjects playing the card game cooperatively. At the same time, each subject's EEG data is simultaneously recorded and transmitted via Bluetooth to three separate Android phones, which act as data acquisition systems. The data from the three phones, the keyboard triggers, and the camera are connected using a WLAN router and synchronized using the LSL software on a laptop. Image(s) provided by Servier Medical Art (https://smart.servier.com), licensed under CC BY 4.0 (https://creativecommons.org/licenses/by/4.0/).

Both experiments had a similar design with minor variations from one another. The design was constructed with blocks of two experimental contrasts, a cooperative task and a non-cooperative (a.k.a. individual) task. Each experimental contrast was preceded by a period of pre-activity rest to serve as a baseline to the experimental contrasts. Each block consisted of one round of the individual task and four to six rounds of the cooperative task. All experiment sessions were carried out in a common office space at the University with participants sitting around a round table equally spaced from each other while the experiment operator and data collection console placed in a corner of the room out of sight from the participants. Figure 2 depicts the experiment set-up for both experimental contrasts as well as for the EEG hyperscanning data collection.

#### 2.2.1 Cooperative task

The cooperative task consisted of participants playing a commercially available card game called “The Mind” (Warsch and Freudenreich, 2018), where each participant received five randomly numbered cards from 1 to 50, with the participants aiming to place their cards in sequential order without any verbal or physical communication with the other participants. A trial was considered successful if all participants placed their cards down in the correct order within a 4-min time limit. In contrast, it was considered a failure if any incorrect card was placed out of order, communication occurred within the group during the task, or the task exceeded the time limit. None of the participants had any prior experience playing the game but were given a training period to familiarize themselves with the rules. Preventing verbal and physical communication from occurring produces fewer muscular artifacts and less noise within the EEG data. The layout of the cooperative task is shown in Figure 2B. The baseline for this condition consisted of a 2-min period in which each participant had to look at their card deck placed facing down in front of them. After the baseline period, participants were allowed to pick up their cards and start the game.

#### 2.2.2 Individual task

The individual task consisted of participants solving different paper-based puzzle games. Each participant was given a different puzzle from a choice of Sudoku, Crossword or Word Search. All puzzles were freely sourced from online collections and chosen for a high degree of difficulty to reduce the risk of participants completing the puzzle before the allocated time of 4 min. The allocation of puzzle game to each participant was done at random in each block, with the constraint that all three puzzles had to be of a different type. The layout of the individual task is shown in Figure 2A. The baseline for this condition consisted of a 2-min period in which each participant had to look at the back side of their puzzle game placed in front of them. After the baseline period, participants were allowed to flip over the paper and the puzzle.

#### 2.2.3 Experiment One vs. Experiment Two

Experiment One consisted of two groups of three participants completing four experiment blocks. Each experiment block consisted of one round of the individual task and four rounds of the cooperative task, yielding eight instances of individual task, 32 instances of cooperative task, and 40 instances of baseline periods. All instances of the baseline period and the individual task had a duration of 2 and 4 min, respectively. However, the cooperative task duration varied depending on whether the group successfully completed (i.e., passed) or failed the game within the allocated 4-min time limit.

Experiment Two consisted of 10 groups of three participants completing one experiment block. Each experiment block consisted of one round of the individual task and six rounds of the cooperative task, yielding 10 instances of the individual task, 60 instances of cooperative task, and 70 instances of baseline periods. In contrast to Experiment One, modifications to the game play were made to ensure that all instances of the individual task and the cooperative tasks were of equal time duration (i.e., 4 min). If the group completed the cooperative task (pass or fail) before the time limit, a new set of cards would be distributed to allow the game play to continue. The decision of whether the trial was a pass or a fail was given by the overall success of the trial, i.e., pass if most of the time the team was successful or failed if most of the time the team was not successful at ordering the given deck of cards. Experiment Two also saw the inclusion of an adversary role in the team. In three of the six cooperative rounds, one of the group members was asked in secret to forfeit the game by placing the incorrect card.

In all six cooperative rounds, each participant was privately provided with written instructions and given 30 s to read them, ensuring that differences in instruction length did not reveal the presence of an experimental manipulation. In fully cooperative rounds, all participants received a brief reminder of the game rules. In adversary trials, one participant was privately instructed to intentionally act against the group's goal by placing an incorrect card at a time of their choosing. The adversary was explicitly instructed to behave as naturally as possible and not to make their actions obvious to the other participants; accordingly, they were not required to place an incorrect card on every turn, nor at a predetermined point in the game. This approach was adopted to preserve the naturalistic character of the task and to avoid overt behavioral cues that could reveal the adversary role. The same participant retained the adversary role across all adversary trials within a given block, such that the remaining two participants were unaware that intentional sabotage was a possible experimental manipulation. Post-experiment debriefing confirmed that participants did not report suspecting purposeful sabotage by another group member.

### 2.3 EEG hyperscanning data acquisition

Each of the three participants in the group wore an mBrainTrain SMARTING mobi EEG system, which are research grade, wireless, wet electrode EEG recording devices. The EEG cap has 24 recording channels placed according to the international 10/20 electrode location standard and with reference and DRL electrodes located at the FCz and Fpz locations respectively. Electrode impedance values were kept below 20 kΩ and preferably below 10 kΩ throughout the experiments to ensure data quality. A keyboard event marker system was implemented to mark the time when events occurred and to ensure that the task start and end times were synchronized across the different data streams. With no specific design consideration, the sampling rate of the EEG data acquisition was 250 Hz for Experiment One and 500 Hz for Experiment Two. A video recording of the game play area was also included in the data collection to evaluate the degree of arm movement in each trial, with the camera being angled downwards to avoid recording the participants' faces, protecting their anonymity. However, the video recordings failed on multiple occasions and were thus not used for further analysis.

Lab Streaming Layer (LSL) (Kothe et al., 2025) was utilized to synchronize the three EEG data streams and the event markers in real-time. Each EEG cap was connected to a mobile recording device via Bluetooth, which was utilized instead of wired cables as wireless produces less movement than wired cable connections. Each device was connected to the same wireless local area network (WLAN), which enabled LSL to record the data simultaneously. This hyperscanning set-up is shown in Figure 2C.

### 2.4 EEG data processing

Given the experimental differences between Experiment One and Two, the preprocessing and analysis were kept separate from each other, and no comparisons were drawn across the two results.

Due to the nature of the data, which is noisy due to the movement required while placing cards during the cooperative task, it was decided not to utilize automatic preprocessing pipelines and to have more control over the rejection of components. Channels and components were manually rejected throughout the entirety of the preprocessing stage. Eliminating these artifacts would also ensure that they are not influencing any neural synchrony observed in the triad. The preprocessing pipeline was kept the same for uniformity and comparisons across trials and groups.

The data were preprocessed in MATLAB (R2023a) (MathWorks, 2023) using the EEGLAB toolbox v2023.1 (Delorme and Makeig, 2004). The three data streams and event markers were imported simultaneously into EEGLAB and synchronized via the MoBILAB plugin (Ojeda et al., 2014). The data were bandpass filtered from 0.1 Hz to 45 Hz using a basic finite impulse response (FIR) filter to remove line noise and DC components. The combined three-stream dataset of 72 channels was then separated into each participant's synchronized EEG data. Each dataset was visually inspected to see if any channels were flat (i.e., no activity was recorded from that specific electrode throughout the experiment). If identified, these channels were removed from the datasets and noted down. Flat channels were removed to allow for full-rank average referencing, independent component analysis, and component removal. Extreme channels, which had amplitudes in the millivolts as opposed to the normal microvolts, were removed as they would contaminate the average referencing and provide inaccurate data representation.

A full-rank average reference was performed on the datasets using the fullRankAveRef plugin (Makoto, 2017), which preserves the data rank while referencing (Klug et al., 2024). The datasets were decomposed into individual components via Independent Component Analysis (ICA) on each dataset. The preprocessing was exhaustive in removing any muscle artifacts or eyeblinks components from the ICA that occurred throughout the trials. For Experiment One, the same four channels were consistently flat or noisy throughout the eight experiments: the occipital electrodes (O1, O2), the central electrode (Cz), and the parietal electrode (Pz). These four channels were removed from the entire study for uniformity in the analysis, which provided 20 channels after completing the preprocessing. For Experiment Two, there was more variation in the number of flat or bad channels, so a zero-column vector was added in place of the flat channels to keep the number of channels within each trial at 24 channels. A total of 3 trials from Experiment One and 18 trials from Experiment Two were rejected due to the data being noisy despite preprocessing or too many flat channels within the dataset, resulting in 267 trials between the two experiments for the analysis.

### 2.5 ISC calculation

Quantifying the IBS between the participants involves using the inter-subject correlation (ISC) analysis method outlined in (Cohen and Parra, 2016) to extract authentic neural responses across trials and subjects. ISC uses correlated component analysis (CorrCA) to identify combinations of neural signals consistently conveyed across numerous trials and subjects by detecting the maximally correlated components between subjects (Parra et al., 2019). Since recorded brain activity is spread over electrodes, CorrCA aims to determine the linear combinations of electrodes with a similar time course in all subjects. CorrCA records the dimensions between subjects with maximum correlation. The analysis method calculates directions in the dimensional space where the data is maximally correlated between subjects with correlation measured across samples (Parra et al., 2019). The final ISC score was calculated as the sum of the three maximally correlated components between the triad for all three experimental contrasts: baseline (a.k.a. resting state), individual task and cooperative task. It is confined to the three greatest components so that the neural measures reflect the levels of synchrony elicited during the task irrespective of the anatomical source. Greater ISC values are achieved when similar responses are evoked across participants (Cohen and Parra, 2016). To validate that the ISC metric could discriminate true synchronization from spurious results, we included a randomized condition by aggregating ISC values obtained from sets of cooperative task EEG recordings from three participants who were not part of the same group but within the same experiment, without altering their EEG time series. CorrCA was employed to assess IBS over other data analysis methods, such as Wavelet Transform Coherence (WTC), due to its ability to record trial variance without requiring predefined frequency bands. Our analysis was interested in understanding neural dynamics from a broadband perspective. ISC using CorrCA is a better method to address this in the absence of a specific hypothesis targeting discrete frequency bands.

#### 2.5.1 Analysis time window

ISC analyses are designed to capture shared neural dynamics elicited by complex, continuous tasks that unfold over extended periods of time, typically on the order of minutes rather than brief, discrete events (Nastase et al., 2019). Consequently, sufficiently long analysis windows are required to reliably estimate inter-brain synchrony while preserving the temporal structure of the task. Consistent with this methodological framework, prior EEG-based ISC studies commonly employ continuous data segments lasting multiple minutes to ensure stable and interpretable correlation estimates during naturalistic and cognitively engaging paradigms (Dauer et al., 2021). Such windows allow intersubject synchrony to emerge from sustained engagement rather than transient or event-locked neural responses. Recent work further supports the use of minute-scale analysis windows in EEG ISC, demonstrating that reliable intersubject synchrony reflects slowly varying cognitive and affective processes that evolve over continuous stimulus presentation (Ueno and Shimada, 2023).

Previous preliminary analysis from our group (Farrell and Valdes, 2023) showed that higher levels of ISC can be observed in shorter analysis time windows. Therefore, it is important to consider this parameter when calculating and interpreting ISC results across contrasts and experiments. Given the experimental design differences from the two experiments in relation to how the cooperative tasks were terminated, Experiment One's analysis time window was set at 60 s for all experimental contrasts while Experiment Two's analysis time window was set at 120 s for all experimental contrasts. Since Experiment One trials were terminated immediately upon a pass/fail event occurring during the cooperative task, individual trial durations varied in length, with the failed task being too short for inclusion and therefore were excluded from the analysis. In contrast, Experiment Two was explicitly designed to ensure equal trial durations across all conditions, thereby enabling direct comparison between cooperative and individual task trials without confounding effects of unequal time windows.

### 2.6 Statistical analysis

Linear mixed-effect models were used to assess the magnitude of differences in ISC scores between conditions in each experiment. The cooperative condition was set as the reference condition for all comparative analyses. Two models were fit for each experiment: the first was unadjusted (A), the second included trial duration as a covariate to account for potential confounding effects of trial duration on computed ISC scores (B). Participant groups were included as random intercepts to account for the lack of independence of measurements within groups across multiple trials.

Homoscedasticity and linearity of data for each experiment were assessed using a Residuals vs. Fitted plot (Supplementary Figures 1, 2). Normality of residuals were assessed using Q-Q plots (Supplementary Figures 3, 4), a correlation plot of experimental duration vs. ISC for Experiment One (Supplementary Figure 5) and a Shapiro-Wilk test (Supplementary Table 1). Experiment One residuals violated assumptions of normality (W = 0.891, *p* < 0.001), however studies have indicated that mixed models are robust to non-normal residuals (Schielzeth et al., 2020). All assumptions were satisfied for Experiment Two. All comparisons between conditions within each experiment were corrected for multiple comparisons using a Bonferroni adjustment, and degrees of freedom were estimated using a Kenward-Roger approximation.

Comparing Pass vs. Fail and Adversary vs. Non-adversary conditions in Experiment Two, ISC scores were averaged within each participant group. Due to unequal distributions (*n* = 10 Pass, *n* = 35 Fail; *n* = 18 Adversary, *n* = 27 Non-adversary), Mann-Whitney U tests were used for these between-group comparisons. All analyses were conducted in R version 4.1.1 (R Core Team, 2021) with a significance threshold set at *p* < 0.05.

## 3 Results

### 3.1 Experiment One

**Figure 3:**
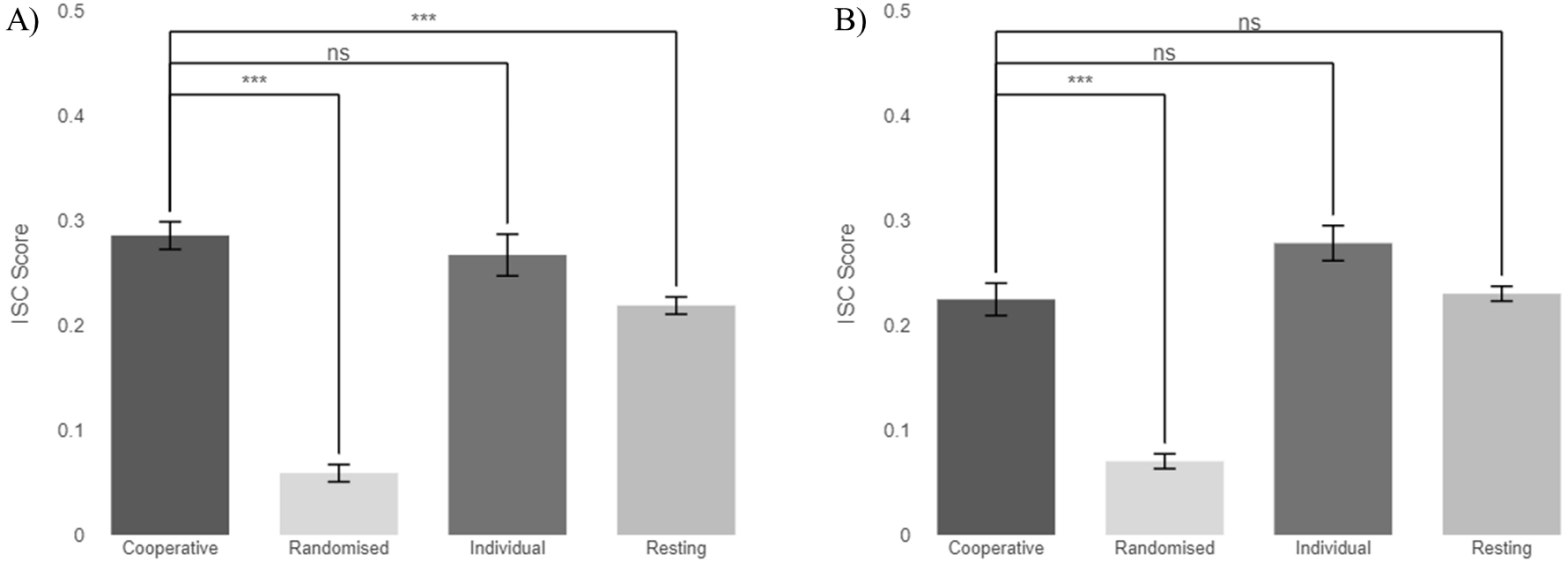
ISC across cooperative tasks, resting state, individual tasks, and randomized data for Experiment One. **(A)** Unadjusted model: differences between cooperative and individual tasks were not significant (*p* = 1.000), while differences between cooperative tasks and the resting and randomized conditions were significantly different (*p* < 0.001). **(B)** Duration-adjusted model: differences between cooperative and individual tasks and resting were not significant (*p* = 0.077 and 1.000, respectively), while differences between cooperative tasks and randomized conditions were significantly different (*p* < 0.001). ns, not significant; ***p < 0.001.

Figure 3 below, depicts the degree of similarity of neural activity within the two triads in Experiment One for the cooperative task and three comparisons: the resting state, the individual task, and randomized data. Figure 3A shows the unadjusted linear mixed-effect model and Figure 3B the duration-adjusted linear mixed-effect model.

The findings on Figure 3A show that there is a similar ISC score for the cooperative task (0.286 ± 0.013) and the individual task (0.267 ± 0.02) compared to the resting state (0.219 ± 0.008) and randomized data (0.059 ± 0.008), supporting the hypothesis that comparable tasks experience similar levels of neural synchrony. In Figure 3B the change in ISC score for the cooperative task is due to the standardization of the duration of the task, which varied among the experimental blocks. The drop in significance is a result of removing the variance induced by duration. The ISC scores differ for the comparisons in this model, with the individual task (0.278 ± 0.017) being greater than resting state (0.23 ± 0.007), cooperative task (0.225 ± 0.015), and randomized (0.07 ± 0.007). Statistical results of Experiment One from the unadjusted model can be seen in Table 1, and from the duration-adjusted model can be seen in Table 2.

**Table 1:** Pairwise comparisons of ISC between conditions from the unadjusted mixed-effects model in Experiment One.

Condition	Estimate	Standardized estimate	df	*t*	*p-*value
Cooperative vs. individual	0.019	0.024	85.156	0.78	*p =* 1.000
Cooperative vs. randomized	0.227	0.016	85.481	14.604	***p** **<*** **0.001**
Cooperative vs. resting	0.067	0.106	85.481	4.304	***p** **<*** **0.001**

Significant values are highlighted in bold.

**Table 2:** Pairwise comparisons of ISC between conditions from the mixed-effects model in Experiment One including duration as a covariate.

Condition	Estimate	Standardized estimate	df	*t*	*p-*value
Cooperative vs. individual	−0.054	0.024	84.953	−2.272	*p =* 0.077
Cooperative vs. randomized	0.154	0.018	83.213	8.475	***p** **<*** **0.001**
Cooperative vs. resting	−0.005	0.018	83.213	−0.297	*p =* 1.000

Significant values are highlighted in bold.

The unadjusted model in Table 1 reveals significant differences between the cooperative task and both resting and randomized conditions, with *p*-values < 0.001. There was no statistical difference between the cooperative and the individual tasks. Table 2, the duration-adjusted model, shows only a significant difference between the cooperative task and randomized data with a *p*-value < 0.001; the other two conditions were non-significant.

### 3.2 Experiment Two

**Figure 4:**
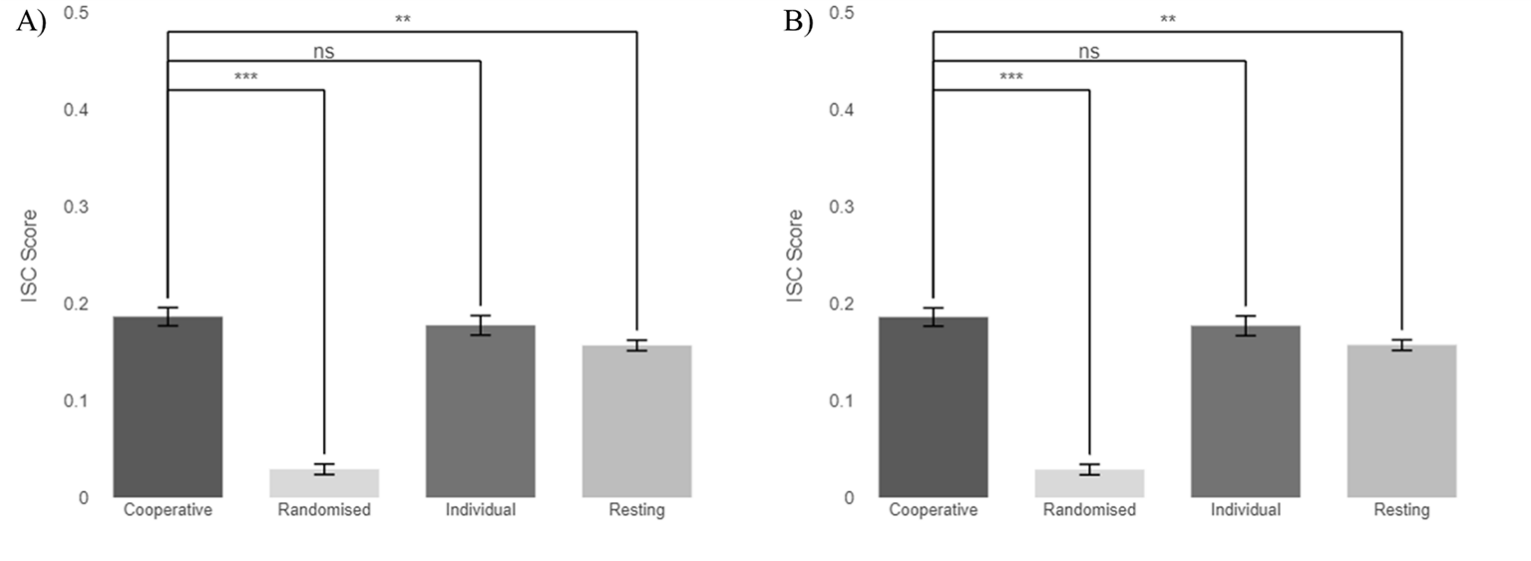
ISC across cooperative tasks, resting state, individual tasks, and randomized data for Experiment Two. **(A)** Unadjusted model: differences between cooperative and individual tasks were not significant (*p* = 1.000), while differences between cooperative tasks and the resting and randomized conditions were significantly different (*p* = 0.004 and < 0.001 respectively). **(B)** Duration-adjusted model: differences between cooperative and non-cooperative tasks were not significant (*p* = 1.000) while differences between cooperative tasks and resting and randomized conditions were significantly different (*p* = 0.007 and < 0.001 respectively). ns, not significant; ***p < 0.001; **p < 0.01.

Figure 4 below, presents the correlation in neural activity observed within the 10 triads during Experiment Two for the cooperative task and three comparisons: the resting state, the individual task, and randomized data. Figure 4A shows the unadjusted linear mixed-effect model and Figure 4B the duration-adjusted linear mixed-effect model.

Similar to the observed findings Experiment One, in the unadjusted model, the cooperative task (0.186 ± 0.009) had a similar value to the individual task (0.177 ± 0.01), with the resting state and randomized data having values of 0.157 ± 0.005 and 0.029 ± 0.005, respectively. In the duration-adjusted model, there was a slight variation in the change in ISC scores as the duration of the tasks was similar across contrasts and experimental blocks. The ISC scores for the comparisons in this model had the same values of (0.186 ± 0.009) for the cooperative task, (0.177 ± 0.01) for the individual task, (0.157 ± 0.005) for the resting state, and (0.029 ± 0.005) for randomized data. Statistical results for Experiment Two from the unadjusted model can be seen in Table 3, and from the duration-adjusted model can be seen in Table 4.

**Table 3:** Pairwise comparisons of ISC between conditions from the unadjusted mixed-effects model in Experiment Two.

Condition	Estimate	Standardized estimate	df	*t*	*p-*value
Cooperative vs. individual	0.009	0.012	113.518	0.719	*p =* 1.000
Cooperative vs. randomized	0.157	0.009	114.246	17.269	***p** **<*** **0.001**
Cooperative vs. resting	0.030	0.009	114.246	3.258	***p** **=*** **0.004**

Significant values are highlighted in bold.

**Table 4:** Pairwise comparisons of ISC between conditions from the mixed-effects model in Experiment Two including duration as a covariate.

Condition	Estimate	Standardized estimate	df	*t*	*p-*value
Cooperative vs. individual	0.009	0.012	112.566	0.724	*p =* 1.000
Cooperative vs. randomized	0.157	0.009	113.318	17.236	***p** **<*** **0.001**
Cooperative vs. resting	0.029	0.009	113.349	3.136	***p** **=*** **0.007**

Significant values are highlighted in bold.

The unadjusted model in Table 3 reveals significant differences for Experiment One between the cooperative task and resting and randomized conditions, with *p*-values = 0.004 and < 0.001, respectively. There was no statistical difference between the cooperative and the individual tasks. Table 4, the adjusted model, saw similar results as the unadjusted model, with significant differences between the cooperative task and both resting and randomized conditions with *p*-values = 0.007 and < 0.001, respectively, but not for the individual task.

#### 3.2.1 Differences in cooperative ISC outcomes: pass vs. fail and adversary vs. non-adversary

**Figure 5:**
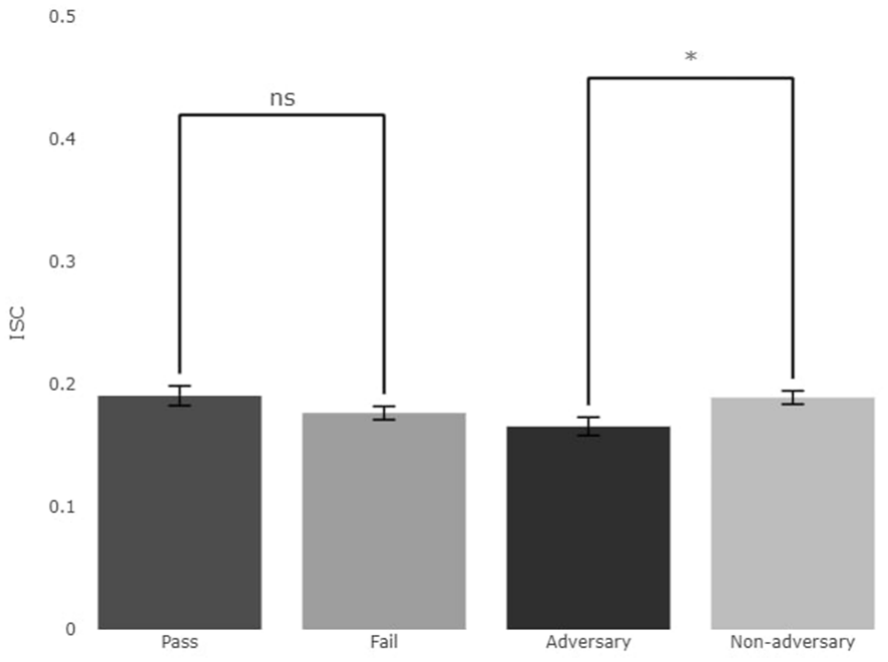
ISC across Experiment Two's comparisons, pass vs. fail results, and the adversary vs. non-adversary results. Differences between passed vs. failed trials were not significant (*p* = 0.189), while differences between adversary vs. non-adversary conditions were statistically significant (*p* = 0.013) with higher ISC values for the non-adversary condition. ns, not significant; *p < 0.05.

Figure 5 below presents the ISC observed during Experiment Two's cooperative task contrasts (i.e., pass vs. fail and adversary vs. non-adversary trial).

The results show marginal differences between the two contrast pairs, with pass slightly performing better than the failed trials with 0.191 compared to 0.177, and non-adversary outscoring adversary with 0.189–0.166. Due to the unequal sample sizes, a Mann-Whitney U test was performed to evaluate the significance of differences between pass vs. fail and adversary vs. non-adversary conditions. The results of this test are shown in Table 5. No significant difference was observed between pass and fail trials (*p* = 0.189). However, a significant difference was observed between mean ISC scores in adversary and non-adversary trials (*p* = 0.013).

**Table 5:** Mann-Whitney U test for Experiment Two within cooperative contrast differences.

Contrasts of interest	U-statistic	*p-*value
Pass vs. fail	224	*p =* 0.189
Adversary vs. non-adversary	137	***p** **=*** **0.013**

Significant values are highlighted in bold.

## 4 Discussion and conclusion

This study aimed to analyze a naturalistic experimental set-up that measured inter-brain synchrony during a three person (triadic) group and individual task at a more comprehensive level to investigate and understand the complex mechanisms exhibited during group behavior across two experiments. Our interest focused on investigating the effect of IBS while participants performed cooperative and non-cooperative tasks in two different scenarios. In Experiment One, the same two groups performed the tasks repeatedly in four blocks. In contrast, in Experiment Two, 10 different groups completed only one block of tasks. To keep the experiment as naturalistic as possible, we conducted the experiment sessions in a common office space where the participants could feel more like in a game session with friends rather than in a laboratory, we also sourced commonly used games and puzzles to provide a sense of normalcy to the session and therefore keeping a higher degree of ecological validity (Vallet, 2024).

To promote cooperative behavior in the experiment, a commercially available card game, whose rules around communication and turn-taking were beneficial for the EEG recordings, was selected. The requirement of no verbal or physical communication between participants, who were strangers to each other, allowed them to formulate strategies to complete the task and encourage cooperative behavior. Strangers were chosen as participants to avoid having to account for varying levels of familiarity between the trials if some of the participants knew each other beforehand.

The hypothesis that an increase in ISC levels for the cooperative task compared to the other conditions would still be present after accounting for confounding artifacts associated with participant behavior (e.g., eyeblinks, eye movements, head movements and muscle components) was partially confirmed. When we don't adjust for experimental duration, as in Figure 3A, the cooperative task's ISC score was similar to the individual task and resting state, but significantly greater than randomized data, indicating similar neural synchrony levels observed during all tasks, with non-statistical significances among the tasks being observed. When adjusting to control for duration as in Figure 3B, different ISC scores are observed, with a smaller cooperative value and individual task value but a larger resting value and randomized. The difference in score between the cooperative task, the resting state, and the individual task compared to the randomized data, given that eyeblinks and muscle artifacts were removed during the preprocessing, suggests that the participants' demeanor does not drive the results (e.g., head movements or gestures); rather, there are underlying neural mechanisms that are evoked due to the commonality of these tasks.

Previous examination of Experiment One data in (Farrell and Valdes, 2023; Kennedy and Valdes, 2024) found greater values of ISC for the resting state compared to the individual task, which could be attributed to the open-eyed resting state being recorded as eliciting similar results across subjects (Olejarczyk et al., 2017; Cohen, 2018). That interpretation suggested that either the different individual tasks assigned to the three individuals contributed to a lower ISC value, or that the common act of sitting still contributed to some brain synchronization among the group. However, the analysis time window parameter was not taken into consideration at the time, and the different contrasts were analyzed with different time windows. Here, in contrast, the findings differ when looking at time-windowed data analysis in Experiment Two, as seen in Figures 4A, B and Figure 5., where the ISC values were slightly lower across all tasks than in Experiment One.

Two theories have been previously proposed in (Hu et al., 2018) for inter-brain synchrony during decision-making, the cooperative interaction hypothesis and the comparable task hypothesis. There was evidence of the cooperative interaction hypothesis in (Farrell and Valdes, 2023; Kennedy and Valdes, 2024), with cooperative task results achieving statistically greater results than other conditions. This paper, however, analyzed the data in a new light, adjusting the trial length to be the same across conditions, finding comparable ISC scores between the cooperative task and the individual task across both experiments and models, see Figures 3, 4. These results suggest that, contrary to the previous interpretation, Hu's second hypothesis is more applicable. The commonality of the individual tasks was enough to see a comparable result to the cooperative task. In our experiment set-up, while each participant received a different puzzle each time, all three types of puzzles belong to the category of “brain teasers” which may be deemed to exert similar cognitive neuronal processes that may in turn drive ISC values higher than hypothesized. Subtle differences between Experiment One and Experiment Two allow us to explore different dimensions of IBS. While not formally reported in this study, evolution of ISC across different blocks of experiments repeated by the same triads in Experiment One did not show evident trends in bonding within the group, increasing ISC score or decline in attention levels due to fatigue from affecting the results, or learning of the card game mechanics as a result of multiple repetitions. In a similar note, no evident triad performance outlier was seen in Experiment Two across the 10 different groups.

In addition to correcting for the cooperative task's effective game time, the other aspect of Experiment Two that differed from Experiment One was introducing an adversary role into half of the trials, where a participant at random was assigned to forfeit the game by misplacing an incorrect card out of order to prompt negative social behavior in the group. In these trials, the adversary was instructed to place an incorrect card at a self-selected time and was not required to sabotage every card placement, in order to avoid overtly revealing their role. These changes to the experiment design allowed us to investigate different elements within the cooperative task, namely, the level of ISC exhibited during passed and failed trials, and the effect of acting against the group's goal. Our findings suggest that while there are no statistical differences between passed and failed trials, there is an effect in brain synchronization driven by the inclusion of an adversary. Cooperative trials with no adversary role had a significantly higher level of synchronization than trials with an adversary in the group.

It is possible that some participants could detect the presence of an adversary within the triad, as the adversary did not change from participant to participant, either due to the lack of subtly from the adversary or the constant misunderstanding of the game, resulting in negative social behavior produced by the triad during both conditions. However, post-experiment debriefing indicated that participants generally did not notice intentional sabotage from another group member, suggesting that incorrect plays were more likely interpreted as errors rather than deliberate opposition. Alternatively, it could indicate a lack of cohesion in understanding the game, as repeated failed trials may have led to frustration and demoralization within the group, explaining the lack of differences across both cooperative and adversary trials in the experiments.

Why is an increase in ISC observed during the cooperative task? In the experimental design, verbal and physical communication between the triad was limited; none of the participants had prior knowledge of each other or the card game. Eye movements and muscle movements were removed through ICA during the data preprocessing, which were not seen to have an impact on neural synchrony; furthermore, as mentioned above, the card game's group bonding, participant attention span, and learning effect of the game did not affect the neural synchrony between the participants. Therefore, some other underlying mechanisms involved in cooperative behavior caused the increase in neural synchrony during prosocial behavior between the group.

Throughout the task, the subjects are confined to looking at each other, where bodily cues can indicate potential turn-taking. Despite limiting physical movement throughout the trials, arm and hand movements when placing down the cards are essential for performing the game correctly. Potentially, these actions could promote some level of synchrony between the subjects. Since other forms of communication and gestures were prohibited during the Experiments, looking at each other was the only interaction between participants. Eye gaze between pairs has been observed to enhance neural synchrony between brains, as discovered during an adult-infant study (Leong et al., 2017). A recent study investigating eye contact's effect on brain synchronization in a social aspect established clear evidence that eye contact promotes greater inter-neural synchrony between pairs and that connections within the brain were susceptible to eye contact in social interactions, discovering that eye contact is intrinsically a communicative cue (Luft et al., 2022). Shared attention is vital in cooperative behavior as the subject must work toward a common goal, where mutual inter-individual eye gazes provide a non-verbal form of communication through turn-taking. This mutual gaze is viewed as shared attention aimed at another individual (Sadato, 2017). Therefore, since eye contact between the triads is a form of communication permitted during the 4 min of interaction, it is a possible source of increased neural synchrony observed between the participants compared to randomized data.

ISC results were greater for Experiment One than Experiment Two across all contrasts, where the experimental duration was shorter, 60 s compared to 120 s. The durations across Experiment One also varied across trials due to card game failures and the recordings being stopped. Supplementary Figure 5, a plot of ISC score vs. time of experiments in seconds results in a statistically significant correlation (*p* = 0.001, *r* = 0.5996), indicating that shorter experimental durations results in greater ISC scores and that the experimental design choice will influence the neural synchrony outcome.

Completing the task requires cooperation, so the subjects must engage with each other through unconscious processes. To determine when it is their turn to place a card correctly, players must “mind read” the other players using their unconscious social intuition by deciding the correct time for playing a card through social cues through the emotional readings of faces or determining the players' intention through eye gazes. ISC captured and quantified the neural synchrony between the subjects; however, it does not fully explain the underlying mechanisms that drive the synchrony; it is simply a summary infographic that determines whether neural synchrony occurred. Further analysis should aim at investigating how theories of the underlying mechanisms in inter-individual connections mentioned above could be determined, represented, or explained. While our study presents an experimental framework for investigating IBS in groups of more than two individuals during cooperative and non-cooperative tasks, there are limitations that need to be considered to prevent the over-interpretation of our findings. This study did not collect any qualitative data in relation to the group composition, personal traits of sociability of the participants, or task preference (i.e., do participants enjoy games or not). Therefore, there is a possibility that confounding effects modulating ISC have not been considered. The lack of categorical differences of the individual task (i.e., brain teaser puzzles) is also a limitation that prevents us from isolating ISC effects to a cooperative and non-cooperative behavior in both Experiments. Further studies in this field should further restrict and limit potential synchrony factors from influencing inter-group neural synchrony, such as eye tracking, and consider the impact that experimental duration will have on results, to be able to begin to explain the complexity of the unconscious processes that occur in individuals during social behavior.

## Data Availability

The raw data supporting the conclusions of this article will be made available by the authors, without undue reservation.
