# Supplementary material for: Analysis of inter-brain synchrony in group-based electroencephalography to assess task-dependent interactions
Source: Front Neuroergon. 2026 Mar 31;7:1774423. doi: 10.3389/fnrgo.2026.1774423 (PMC13076319; doi:10.3389/fnrgo.2026.1774423)
Supplement: Supplementary file 1 [file Data_Sheet_1.docx]

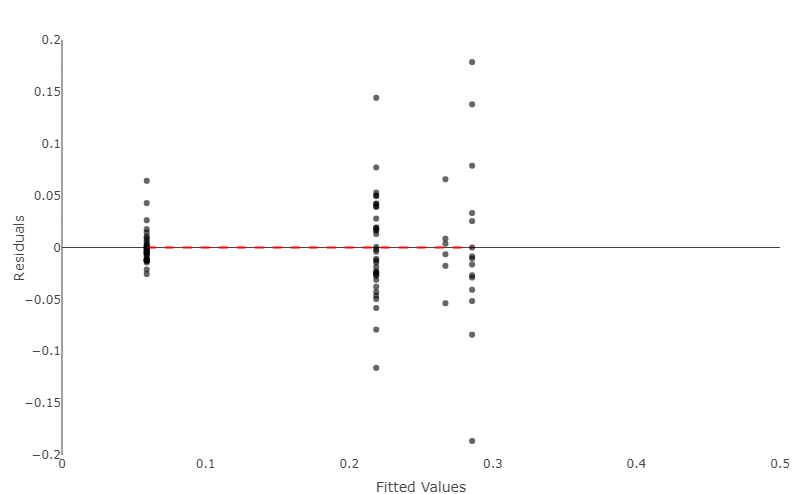
**Supplementary Figure 1 -** Residuals versus fitted values from the unadjusted mixed-effects ISC model for Experiment One


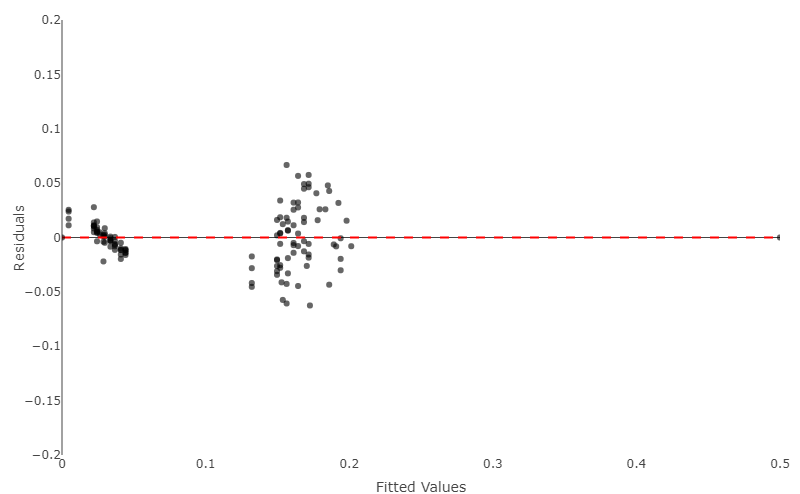


**Supplementary Figure 2 -** Residuals versus fitted values from the unadjusted mixed-effects ISC model for Experiment Two


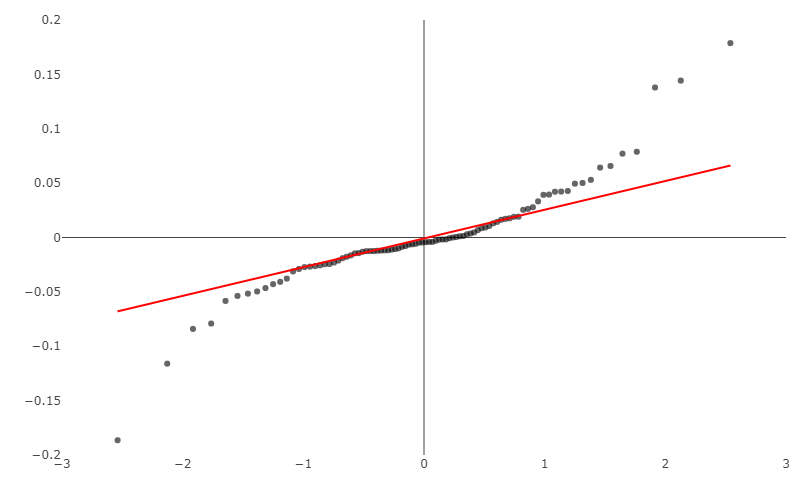
**Supplementary Figure 3 -** Q–Q plot of residuals from the unadjusted mixed-effects ISC model for Experiment One


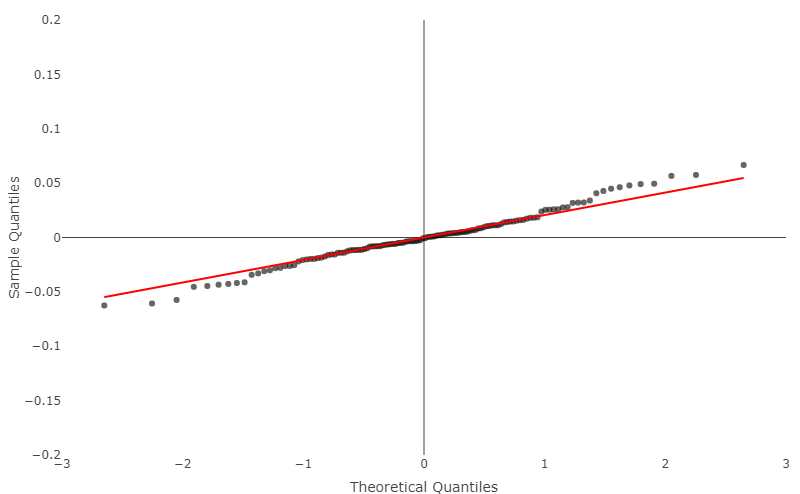
**Supplementary Figure 4 -** Q–Q plot of residuals from the unadjusted mixed-effects ISC model for Experiment Two



**Supplementary Figure 5 -** Duration versus ISC for unadjusted model in Experiment One

**Supplementary Table 1: Shapiro-Wilk Normality Tests**

| **Experiment** | **W-value** | **p-value** |
| --- | --- | --- |
| Experiment 1 | .896 | **p < 0.001** |
| Experiment 2 | .987 | 0.261 |

Note: Significant p-values are highlighted in bold.
